# The earliest trough concentration predicts the dose of tacrolimus required for remission induction therapy in ulcerative colitis patients

**DOI:** 10.1186/s12876-015-0285-3

**Published:** 2015-04-29

**Authors:** Sakiko Hiraoka, Jun Kato, Yuki Moritou, Daisuke Takei, Toshihiro Inokuchi, Asuka Nakarai, Sakuma Takahashi, Keita Harada, Hiroyuki Okada, Kazuhide Yamamoto

**Affiliations:** 1 Department of Gastroenterology and Hepatology, Okayama University Graduate School of Medicine, Dentistry and Pharmaceutical Sciences, 2-5-1 Shikata-cho, Kita-ku, Okayama, 700-8558 Japan; 2 Second Department of Internal Medicine, Wakayama Medical University, Wakayama, Japan

**Keywords:** Ulcerative colitis, Tacrolimus, Single-nucleotide polymorphisms, Remission

## Abstract

**Background:**

Oral tacrolimus therapy is effective for refractory ulcerative colitis (UC), but dose adjustment according to the trough concentrations which varies largely among individuals, is required. This study aimed to identify factors to predict the tacrolimus dose required for achieving the target trough level for remission induction of UC.

**Methods:**

Forty-seven consecutive UC patients who were treated with tacrolimus were retrospectively analyzed. Tacrolimus doses were adjusted every 2 or 3 days to achieve trough concentrations of 10–15 ng/mL. The dose required for reaching the target trough level was analyzed based on disease characteristics, course of trough concentrations, and gene polymorphism related to tacrolimus metabolism.

**Results:**

Median daily dose of tacrolimus required for achieving the target trough level was 0.19 (0.07-0.42) mg/kg, and patients were divided into high or low dose group (< 0.2 mg/kg or > 0.2 mg/kg). The value of initial trough concentration/starting dose was higher in the low dose group than in the high dose group (1.35 ng/mL/mg vs. 0.78 ng/mL/mg, *p* < 0.0001). Although presence of *CYP3A5* *1 was more frequently observed in the high dose group, initial trough concentration was the only significant factor for determining requirement of high dose of tacrolimus (OR = 28.0, 95% confidence interval 3.20 – 631).

**Conclusions:**

The most practical predictor of the dose required for achieving the target trough concentration was the trough concentration measured 2 or 3 days after starting tacrolimus therapy. Our findings would make tarcolimus administration for UC safer, easier and more effective.

## Background

Ulcerative colitis (UC) is an idiopathic chronic inflammatory disorder characterized by manifestations such as diarrhea, rectal bleeding, abdominal pain, fever, anemia, and body weight loss [1]. 5-aminosalicylates (5-ASA) are the basic therapeutic drug, and most patients with mild to moderate UC respond to them. Corticosteroid treatment is considered in patients with more severe symptoms when 5-ASA are not effective. However, intravenous steroids are not effective in 20-30% of patients, and these patients ultimately are likely to require colectomy [2].

Calcineurin inhibitors such as cyclosporine A and tacrolimus have been shown to be effective for treatment of steroid-dependent or steroid-refractory UC patients with moderate to severe activity. Both of the medications were effective for approximately 80% of refractory UC patients [3-7]. To maximize the efficacy, however, these drugs require adjustment of the doses for administration by routinely measuring blood concentrations. To avoid substantial fluctuation in the blood concentrations of cyclosporine, it has to be administered by continuous intravenous infusion. On the other hand, tacrolimus is usually taken orally with sufficient efficacy by monitoring trough blood concentrations of the drug. However, a large variation in the dose required for reaching sufficient blood trough levels among individuals has made the administration of tacrolimus difficult.

Tacrolimus is metabolized primarily by cytochrome P450 3A4 (CYP3A4) and CYP3A5, and converted to a substrate of ATP-binding cassette sub-family B member 1 (ABCB1) [8,9]. It has been reported that single-nucleotide polymorphisms (SNPs) of *CYP3A4*, *CYP3A5*, and *ABCB1* are correlated with altered pharmacokinetics of tacrolimus in patients with organ transplantation [10,11]. Subjects having the *CYP3A5* *1 allele express large amounts of CYP3A5 protein which promote metabolism of tacrolimus [11]. In the treatment of UC, Hirai et al. recently reported that a SNP of *CYP3A5* was correlated with not only the period and sufficient dose required for reaching the target trough concentration, but also efficacy of tacrolimus therapy [12]. In clinical settings, however, it is not practical to examine SNPs before tacrolimus administration because of the cost and clinical unavailability. Moreover, whether patients should be treated with tacrolimus is determined regardless of presence of the SNPs because the treatment options for refractory UC are limited. In this context, easy and practical methods to predict optimal dose of tacrolimus for achieving sufficient trough levels are eagerly anticipated.

In this study, therefore, we meticulously examined the courses of trough concentrations of tacrolimus in UC patients who were administered tacrolimus as remission induction therapy, and aimed to identify predictive factors for the dose of tacrolimus required to achieve sufficient trough levels which could be easily used in actual clinical settings.

## Methods

### Patients and procedures

Data on all patients with moderate to severe UC who were treated with tacrolimus at Okayama University Hospital and Wakayama Medical University Hospital from September 2006 to October 2012, were analyzed. A diagnosis of UC was established by the presence of accepted clinical, endoscopic, and histologic criteria. No patients had any renal function disorders, and if patients received tacrolimus therapy twice or more, only the initial administration episode was included in the analysis. This study was approved by the institutional review board of both institutions. Ethics Committee of Okayama University Graduate School of Medicine, Dentistry and Pharmaceutical Sciences and Wakayama Medical University approved the study protocol including DNA analysis (IRB No.218), and written informed consent was received from each patient. There were no conflicts of interest or sponsors of this study.

Medical charts provided clinical information including demographic data, such as age, gender, and duration of the disease, as well as disease status, such as severity of disease, extent of disease, and medications. According to Truelove and Witts’ criteria [13], all patients had either moderate or severe disease, and all were steroid-dependent or steroid-refractory. Steroid-dependency was defined as the inability to completely withdraw systemic steroids because of disease relapse or the occurrence of at least two flare-ups requiring systemic steroid therapy in a six-month period. Steroid-refractoriness indicated little or no improvement after two weeks of systemic steroid therapy (at least 30 mg prednisolone daily).

As candidates of predictive parameters, concomitant medications (5-ASA, corticosteroids, immunomodulator (azathioprine or 6-mercaptopurine) use), bowel movement (frequency/day), diet (fasting or not), and laboratory data including leukocyte count, platelet count, hemoglobin, and C-reactive protein (CRP) at the start of tacrolimus therapy were specifically examined. Tacrolimus dose was analyzed with adjustment by body weight.

### Administration and monitoring of tacrolimus

For patients who were treated with oral tacrolimus therapy, capsules (Prograf; Astellas Pharma Inc., Tokyo, Japan) containing 0.5 mg or 1 mg of tacrolimus were used. The initial oral dose of tacrolimus was 0.05-0.15 mg/kg/day twice daily. Whole blood trough concentration of tacrolimus was measured at each hospital once every 2–3 days by the affinity column mediated immunoassay. The dose of tacrolimus was adjusted in the evening according to the trough concentration measured in the morning of the same day. The target whole blood trough concentration was 10–15 ng/mL and dose adjustment was done to achieve the target concentration as soon as possible within 2–3 weeks [6,14,15]. For most of cases, adjustment was made to reach the target within one week. The equations for dose adjustment were as follows: previous dose × (10 ng/mL/blood trough concentration) until 3 days after starting tacrolimus, and previous dose × (12.5 ng/mL/blood trough concentration) more than 3 days after starting tacrolimus.

All responding patients were followed by tapered blood trough levels of 5–10 ng/mL for about 3–6 months.

### Evaluation of efficacy of tacrolimus

The efficacy of tacrolimus was evaluated by criteria based on changes in the clinical activity index (CAI) scores proposed by Lichtiger et al [3]. Clinical courses were classified as remission, response, and no response. Remission was defined as a CAI score of 3 or lower within 4 weeks of starting tacrolimus therapy. Response was defined as a CAI score of 4 or higher, but equal to or lower than half the score at the start of tacrolimus therapy. ‘Effective’ case was defined as a patient either in remission or with response. ‘No response’ was defined as a clinical course that was not identified as in remission or with response. All of these definitions were determined regardless of steroid administration status, because relatively short-term effect was evaluated in this study. Adverse events of tacrolimus were counted when reduction of the dose or discontinuation of tacrolimus was needed.

### Analysis of SNPs of genes associated with tacrolimus pharmacokinetics

Genetic analysis of *CYP3A5* and *ABCB1* polymorphisms was performed according to previous reports [16]. In brief, genomic DNA was extracted from peripheral blood using the QiaAmp DNA Mini kit (Qiagen, Valencia, CA, USA). We analyzed the polymorphisms of *CYP3A5* (rs776746) and *ABCB1* exon 26 (rs1045642) genes using TaqMan® SNP Genotyping Assay (Applied Biosystems, Foster City, CA, USA) with a LightCycler 480 system, as recommended by the manufacturer (Roche Diagnostics, Basel, Switzerland).

### Statistical analysis

We used the chi-square test or Fisher’s exact test for univariate analysis and the logistic regression model for multivariate analysis in order to identify factors contributing to the dose of tacrolimus required. Results are shown with calculations for odds ratios (ORs) and 95% confidence intervals (CIs). These analyses were performed using the JMP program (version 8, SAS Institute, Cary, NC, USA). To estimate appropriate cutoff values for age, leukocyte count, platelet count, hemoglobin, and CRP, receiver operating characteristic (ROC) curve analysis was performed. All *p* values were two-sided and considered significant when less than 0.05.

## Results

### Clinical characteristics of patients

We administered tacrolimus to 47 UC patients during the study period. Characteristics of the study population are shown in Table 1. The patients included 24 (51%) men and 23 (49%) women, with a median age of 40 years (range; 12–70 years) at the initial administration of tacrolimus treatment. All patients had either pancolitis or left-side colitis. Forty-two (89%) patients used 5-ASA, 42 (89%) patients used corticosteroids, and 13 (28%) patients used immunomodulators (azathioprine (AZA) or 6-mercaptopurine (6-MP)). The disease duration of the patients was 43.6 (0.74-336) months. Of these patients, 41 (87%) achieved clinical remission or response and 6 (13%) had no response with tacrolimus therapy.

**Table 1:** **Characteristics of study population**

Total	47
Age, years, median (range)	
at onset	30 (11–67)
at initiation of tacrolimus	40 (12–70)
Disease duration, months, median (range)	43.6 (0.74-336)
Gender	
Male	24 (51%)
Female	23 (49%)
Disease severity	
Moderate	34 (72%)
Severe	13 (28%)
Extent of disease	
Pancolitis	34 (72%)
Left-side colitis	13 (28%)
Concomitant therapy	
5-ASA	42 (89%)
Corticosteroid use	42 (89%)
Immunomodulator use	13 (28%)
SNPs analysis	
*CYP3A5* (*1*1/*1*3/*3*3)	1/12/24
*ABCB1* exon26 (CC/CT/TT)	15/14/8

The SNP analysis was performed only for patients who provided written informed consent for this procedure.

SNPs, single-nucleotide polymorphisms 5-ASA, 5-aminosalicylic acid.

*CYP3A5, cytochrome P450 3A4.*

*ABCB1, ATP-binding cassette sub-family B member 1.*

### The dose of tacrolimus required for the target trough level

**Figure 1:**
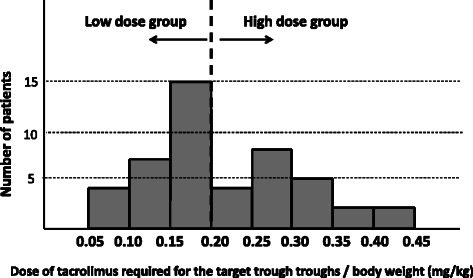
The distribution of the dose required for reaching the target trough concentration of tacrolimus/body weight. The median dose required for reaching the target trough concentration of each patient was 0.19 mg/kg, and the peak of the distribution was 0.15 mg/kg – 0.20 mg/kg.

Figure 1 shows a histogram of the tacrolimus dose required for reaching the target trough concentration (mg)/body weight (kg) of the 47 patients. Median daily dose of tacrolimus required for the target trough level (10–15 ng/mL) was 0.19 (0.07-0.42) mg/kg, and the largest number of patients was distributed within the range from 0.15 to 0.20 mg/kg. Hence, the patients were divided into two groups according to the required dose (low dose group: < 0.2 mg/kg, or high dose group: > 0.2 mg/kg).

Table 2 shows the comparison of clinical characteristics between patients of the low dose group and those of the high dose group. Clinical and medical characteristics did not differ significantly between the two groups. Laboratory data at the start of tacrolimus administration demonstrated that patients of the high dose group tended to have higher CRP than patients of the low dose group (2.20 (0.04-14.7) vs. 0.93 (0.02-7.73), *p* = 0.053). The results of SNP analysis of genes associated with tacrolimus pharmacokinetics indicated that *CYP3A5* *3*3 was significantly more frequently observed in patients of the low dose group than in those of the high dose group (19 (90%) vs 5 (31%), *p* = 0.001).

**Table 2:** **The differences in clinical background between patients of the low dose group and the high dose group**

	Low dose^a^ < 0.2 mg/kg	High dose^a^ ≥ 0.2 mg/kg	Univariate analysis
	n = 26	n = 21	*p*
Median(range) age at onset, years	36 (12–67)	31 (11–64)	NS
Median(range) age at starting tacrolimus, years	42.5 (12–70)	36 (14–67)	NS
Median(range) duration of disease, months	28.7 (0.74-336)	62.2 (0.97-323)	NS
Gender			
Male/Female	10/16	14/7	0.08
Disease severity			
Moderate/Severe	20/6	14/7	NS
Extent of disease			
Pancolitis/Left-side colitis	19/7	15/ 6	NS
Medications			
5-ASA	25	17	0.16
Corticosteroid use	24	28	NS
Dose of corticosteroid at initiation of tacrolimus (PSL)	25 (0–40)	20 (0–30)	0.12
Immunomodulator use	8	5	NS
Bowel movement at initiation of tacrolimus (times/day)	8 (4–20)	7.5 (4–15)	NS
Diet at initiation of tacrolimus			
Fasting	6	6	NS
Laboratory data at initiation of tacrolimus			
White blood cell count (×10^3^/μL)	7.52 (4.71-15.0)	7.50 (5.46-15.2)	NS
Hemoglobin (mg/dL)	11.2 (7.50-13.6)	10.4 (7.90-14.4)	NS
Platelet count (×10^4^/μL)	39.6 (9.70-65.6)	38.1 (12.0-75.2)	NS
CRP (mg/dL)	0.93 (0.02-7.73)	2.20 (0.04-14.7)	0.053
SNPs analysis^b^			
*CYP3A5* (*1*1/*1*3/*3*3)	0/2/19	1/10/5	0.001
*ABCB1* exon26 (CC/CT/TT)	9/8/4	6/6/4	NS

^a^Patients were divided into two groups by the maximum dose (<0.2 mg/kg or ≥ 0.2 mg/kg).

^b^SNPs analyses were performed in 37 patients.

SNPs, single-nucleotide polymorphisms.

5-ASA, 5-aminosalicylic acid.

CRP, C-reactive protein.

*CYP3A5, cytochrome P450 3A4.*

*ABCB1, ATP-binding cassette sub-family B member 1.*

### Clinical courses of patients in the low dose group vs. high dose group

**Figure 2:**
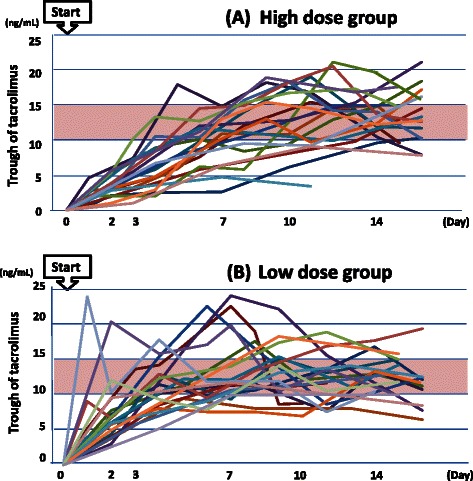
The courses of trough concentrations of tacrolimus in patients belonging to the high-dose group **(A)** and to the low dose group **(B)**. While the trough concentrations of patients in the high dose group increased gradually without outliers, some of the patients in the low dose group showed excessive trough concentrations early after starting tacrolimus.

The time courses of the trough concentrations of the patients in each group are shown in Figure 2. As shown in the figure, while the trough concentrations of patients in the high dose group increased gradually without outliers, some of the patients in the low dose group showed excessive trough concentrations early after starting tacrolimus. In fact, the trough concentrations exceeded 20 ng/mL within 7 days after start of tacrolimus in 5/26 (19%) patients of the low dose group.

Table 3 shows the clinical courses of patients in the low vs. high dose groups. The period required for reaching the target trough concentration was significantly shorter in patients of the low dose group than in those of the high dose group (5 (2–13) days vs. 7 (3–17) days, *p* = 0.011). In addition, the percentage of patients who attained the target trough levels within 7 days was higher in the low dose group (88% vs. 62%, *p* = 0.043). Moreover, the period required for reaching remission was significantly shorter in the low dose group than in the high dose group (14 (5–26) days vs. 18 (13–42) days, *p* =0.0055), although the remission rate did not differ significantly between the two groups. Adverse events that required discontinuation or reduction of the drug occurred slightly more frequently in patients of the low dose group, although statistical significance was not observed (6/26 (23%) vs. 2/21 (10%), *p* = 0.27). Details of the adverse events were nausea (3 patients), renal dysfunction (2 patients), and headache (1 patient) in the low dose group, while nausea (1 patient) and fatigue (1 patient) in the high dose group. Thus, the classification by our criteria (low dose group vs. high dose group) appears to be reasonable, and if it could be predicted into which group a patient is classified, more appropriate dose modification could be tailored to each patient.

**Table 3:** **Differences in the courses of tacrolimus therapy between patients of the high dose group and low dose group**

	Low dose^a^	High dose^a^	*p*
	n = 26	n = 21	
Period required for reaching the target trough level (>10 ng/mL) of tacrolimus (day)	5 (2–13)	7 (3–17) ^b^	0.011^b^
Patients who attained the target trough level (>10 ng/mL) within 7 days	23 (88%)	13 (62%)	0.043
The initial trough concentration/starting dose of tacrolimus (ng/mL/mg)	1.35 (0.50-3.60)	0.78 (0.30-1.30)	<0.0001
Efficacy			
Remission	18 (70%)	12(57%)	NS
Response	4 (15%)	7 (33%)	
No response	4 (15%)	2 (10%)	
Period required for reaching remission (day)	14 (5–26)	17.5 (13–27)	0.0055
Adverse events			
Required for discontinuation and reduce	6 (23%)	2 (10%)	0.27

^a^Patients were divided into two groups by the maximum dose (<0.2 mg/kg or ≥ 0.2 mg/kg).

^b^Two patients of the high dose group could not achieve the trough concentration of 10 ng/mL. The analysis was performed excluding these two patients.

### Predictors of the dose required for achieving the target trough level

As shown in Figure 2, the trough levels measured early from the start of tacrolimus therapy may differentiate between the low dose and high dose groups. In addition, Table 3 indicates that the value of initial trough concentration/starting dose was significantly higher in patients of the low dose group than in those of the high dose group (1.35 (0.50-3.60) vs. 0.78 (0.30-1.30), *p* < 0.0001). Therefore, the early trough concentration, which can be represented as the value of initial trough concentration/starting dose of tacrolimus, is considered to be one of the predictors of the dose required for achieving the target trough level.

**Figure 3:**
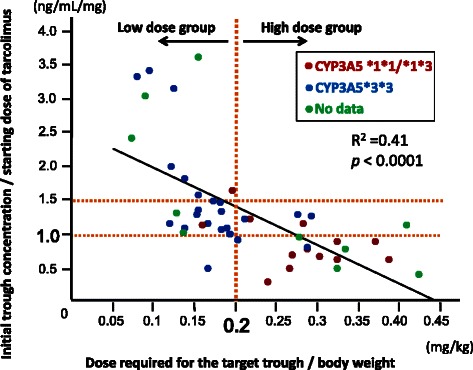
The relationship between initial trough concentrations and doses required for sufficient trough levels. The value of initial trough concentration/starting dose of tacrolimus (ng/mL/mg) and the dose of tacrolimus / body weight (mg/kg) required for reaching the target trough level were inversely correlated (R^2^ = 0.41, *p* < 0.001).

To precisely evaluate the correlation between the initial trough concentration and the required doses for the target trough level, we analyzed 42 patients who underwent initial measurement of the trough concentration at 2 or 3 days after starting tacrolimus. The median initial trough concentration of those patients was 5.3 (0.9-20.5) ng/mL, and the value of initial trough concentration/starting dose of tacrolimus (ng/mL/mg) and the dose of tacrolimus required for the target trough level/body weight (mg/kg) were significantly inversely correlated (R^2^ = 0.41, *p* < 0.0001) (Figure 3). All patients with a value of initial trough concentration/starting dose (ng/mL/mg) > 1.5 were categorized into the low dose group. On the other hand, only one of 17 patients with a value of initial trough concentration/starting tacrolimus dose (ng/mL/mg) < 1.0 was placed into the low dose group. An ROC analysis revealed that the value of initial trough concnentraion/starting dose of 1.0 ng/mL/mg discriminated most precisely between the low and high dose groups (AUC = 0.876). These results suggest that the trough concentration at 2 or 3 days after starting tacrolimus could be an indicator determining whether the patient require high or low doses of tacrolimus.

Meanwhile, as shown in Table 2 and Figure 3, the patients with *CYP3A5* *1 allele (red dot) were more likely to belong to the high dose group, while the patients without this allele (blue dot) were more likely to be classified into the low dose group (presence/absence of *CYP3A5* *1 allele: low dose group, 11/5 vs. high dose group, 2/19; *p* = 0.0003). Therefore, the SNP of *CYP3A5* is another candidate predictor of the tacrolimus dose required for achieving the target trough concentration.

### Predictive values for patients requiring high tacrolimus doses: the initial trough concentration vs. gene polymorphism

As we have shown, two parameters, the initial trough concentration and the SNP of *CYP3A5*, could predict patients requiring high tacrolimus doses. Thus, we compared the predictability between the two parameters using multivariate analysis (Table 4). A value of initial trough concentration/starting dose < 1 ng/mL/mg is a significant predictor of patients requiring high doses of tacrolimus (OR = 60.8, 95%CI 3.20-631), *p* < 0.0001). On the other hand, SNP of *CYP3A5* did not reach statistical significance.

**Table 4:** **Multivariate analysis of factors predictive for requiring high tacrolimus doses (> 0.2 mg/kg)**

	Multivariate analysis	
	*p*	
*CYP3A5* (*1*1 or *1*3)	6.14 (0.61 - 66.8)	0.12
The initial trough concentration/starting dose > 1 ng/mL/mg^a^	28.0 (3.20 - 631)	<0.0001

^a^To estimate appropriate cutoff values, receiver operating characteristic (ROC) curve analysis was performed.

*CYP3A5, cytochrome P450 3A4.*

## Discussion

Both of the calcineurin inhibitors, cyclosporine and tacrolimus, have been shown to be effective for refractory UC [3-7,12,14]. Whereas cyclosporine has to be administered intravenously due to the variable nature of blood concentration, tacrolimus can be used by peroral administration, usually twice a day, with sufficient efficacy. It has been reported that the optimal trough concentration of 10–15 ng/mL should be attained ‘quickly and safely’ for successful treatment. However, the doses required for reaching the target trough level differ largely among patients, and therefore, management of the trough concentrations is often difficult. Delay in reaching the target trough level would also delay treatment response, and may decrease the efficacy of the drug, whereas too rapid increase in blood concentration could cause adverse events. In this study, to ease the administration of tacrolimus, we looked for markers to predict the dose required for achieving the target blood concentration among materials already available in actual clinical practice.

**Figure 4:**
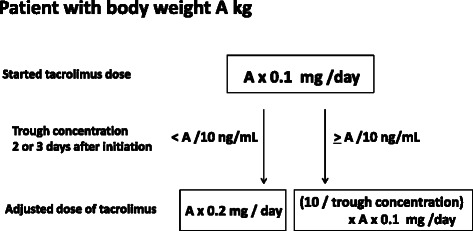
The algorithm based on the earliest trough concentration. If a patient with body weight of 60 kg started tacrolimus therapy with 6 mg/day, and the trough concentration measured 2 or 3 days after initiation was < 6 ng/mL, the dose should be increased to 12 mg/day. On the other hand, if the trough concentration measured 2 or 3 days after initiation was > 6 ng/mL, the increase of the dose should be minimized

It is known that tacrolimus is metabolized primarily by CYP3A4 and CYP3A5, and is converted to a substrate of ABCB1. Hirai et al. previously reported that a SNP of *CYP3A5* affected pharmacokinetics of tacrolimus and the presence of the *CYP3A5* *1 allele was a predictor of requirement of high dose administration. Moreover, they reported that patients without the *CYP3A5* *1 allele were more likely to enter remission with tacrolimus therapy. In line with this report, we also showed that the SNP of *CYP3A5* affected pharmacokinetics of tacrolimus and predicted the requiring dose of the drug to some extent. However, examination of SNPs prior to prescription of the drug is not practical due to the cost, unavailability at hospitals, and ethical problems. Hence, we meticulously analyzed the disease course and drug trough concentrations of the patients who were treated with tacrolimus, and found that the trough concentration measured 2 or 3 days after starting the treatment is a more precise predictor of dose requirement of the drug for remission induction therapy. This predictor is very simple and can be used without any other examinations or parameters. For instance, if a patient with body weight of 60 kg started tacrolimus therapy with 6 mg/day, and the trough concentration measured 2 or 3 days after initiation was < 6 ng/mL, the dose should be increased to 12 mg/day. On the other hand, if the trough concentration measured 2 or 3 days after initiation was > 6 ng/mL, the increase of the dose should be minimized (Figure 4). Thus, our results would be very useful in adjusting administration of tacrolimus for remission induction therapy in UC patients.

Patients with the *CYP3A5* *1 allele are rich in the CYP3A5 protein and can metabolize tacrolimus more efficiently. Therefore, the results regarding the requirement of a higher dose to reach the target trough concentration in patients with *CYP3A5* *1 allele appeared to be consistent and corroborative with those of the previous report by Hirai et al [12]. In contrast to the previous report, however, the remission rate within 4 weeks did not differ significantly between the two groups (13/24 (54%) vs. 10/13 (77%), *p* = 0.29). The discrepancy could be attributable to the following reasons.

First, the patients in the report by Hirai et al. had more severe disease than our cohort; all of their patients were hospitalized, and the average CRP appeared to be more than 5 mg/dL. In contrast, 14/47 (30%) of our patients were outpatients, and the median CRP of our patients was 1.4 mg/dL. Perhaps due to the difference in severity, the total remission rate was higher in our study than in the previous study (30/47 (64%) vs.14/45 (31%)). The higher remission rate of our study was probably one of the contributing factors for lack of statistical significance between patients with and without the SNP. Second, the frequency of adjustment of tacrolimus doses may be different. We measured trough concentrations of tacrolimus once every 2–3 days, while Hirai et al. appeared to measure the trough concentration only twice, on days 2–5 and 7–10, during the early period of therapy. The frequent adjustment of the dose of tacrolimus may have increased the remission rate of our patients. Whether or not our easy method for predicting the dose requirement of tacrolimus is valid in more severe UC patients should be verified in future studies.

It is expected that our results would make the handling of tacrolimus easier and would expand the use of the drug in the clinical situation. Because the treatment tools for refractory UC are still limited, tacrolimus could be a user-friendly option that would impact greatly on the clinical practice for refractory UC patients in several manners. First, while another calcineurin inhibitor, cyclosporine, requires continuous intravenous infusion, tacrolimus can be taken orally, indicating that the drug can be prescribed for outpatients. Our results would make the trough management of tacrolimus therapy for outpatients easier and reduce the frequency of clinic visits, possibly resulting in an increase in the success rate of the treatment. Second, in current clinical situations, anti-tumor necrosis factor alpha antibodies such as infliximab and adalimumab are usually used for refractory UC outpatients. Although such agents have also been shown to be effective for refractory UC patients [17,18], those are injected agents, thus, patients have to visit a clinic or hospital periodically for infusions or perform self-injection. In contrast, tacrolimus has an advantage that it is a peroral drug and easily acceptable for every type of patients. Third, appropriate management of the blood concentration of tacrolimus would reduce adverse events due to the drug. The relatively frequent adverse events of tacrolimus are nausea, headache, tremor, and renal dysfunction. Those events usually occur when blood concentrations of the drug are too high. In particular, nausea and headache are likely to occur when the blood concentration abruptly increases. Reduction of adverse events by appropriate monitoring of the blood concentration as per our results would also lead to increased success of the tacrolimus treatment.

The starting dose of tacrolimus for UC used in the previous phase III study was 0.05 mg/kg/day [6,14]. In this study, the average initial dose of tacrolimus was 0.10 mg/kg/day, although there was variation among patients due to the retrospective cohort. As shown in Figure 1, the dose required for the target trough level was more than 0.10 mg/kg/day in 91% of patients, thus, the initial dose of 0.10 mg/kg/day would be reasonable. In this situation, however, patients of the low dose group more frequently developed adverse events (23%) mostly due to excessive trough concentrations, although most of these events were mild. Therefore, our findings regarding the prediction of requirement of low or high dose within 2 or 3 days after starting tacrolimus would be helpful in view of avoidance of adverse events.

There are limitations to the present study. Because this was a retrospective clinical study, the intervals of trough measurement differed among patients. The initial trough concentration was not measured at 2 or 3 days after start of tacrolimus in several patients, and those patients were not included in the corresponding analyses. Trough management based on our results should be validated in prospective studies in the future. The SNP analysis also could not be performed in a few patients who did not give approval for the analysis.

## Conclusions

We found that the initial trough concentration could be a useful predictor of the dose of tacrolimus required for remission induction therapy. The drug monitoring based on our findings can be utilized in any situation of clinical practice and does not need specific examinations such as analysis of gene polymorphism. Our findings would make tacrolimus therapy for refractory UC patients more patient- and physician-friendly.

## Abbreviations

UC: Ulcerative colitis
5-ASA: 5-aminosalicylates
CYP3A4: Cytochrome P450 3A4
ABCB1: ATP-binding cassette sub-family B member 1
SNPs: Single-nucleotide polymorphisms
CRP: C-reactive protein
CAI: Clinical activity index
ORs: Odds ratios
CIs: Confidence intervals
ROC: Receiver operating characteristic
AZA: Azathioprine
6MP: 6-mercaptopurine

## References

[1] Podolsky DK (2002). Inflammatory bowel disease. N Engl J Med.

[2] Turner D, Walsh CM, Steinhart AH, Griffiths AM (2007). Response to corticosteroids in severe ulcerative colitis: a systematic review of the literature and a meta-regression. Clin Gastroenterol Hepatol.

[3] Lichtiger S, Present DH, Kornbluth A, Gelernt I, Bauer J, Galler G (1994). Cyclosporine in severe ulcerative colitis refractory to steroid therapy. N Engl J Med.

[4] Benson A, Barrett T, Sparberg M, Buchman AL (2008). Efficacy and safety of tacrolimus in refractory ulcerative colitis and Crohn’s disease: a single-center experience. Inflamm Bowel Dis.

[5] Yamamoto S, Nakase H, Mikami S, Inoue S, Yoshino T, Takeda Y (2008). Long-term effect of tacrolimus therapy in patients with refractory ulcerative colitis. Aliment Pharmacol Ther.

[6] Hiraoka S, Kato J, Suzuki H, Yamamoto K (2012). Readministration of calcineurin inhibitors for ulcerative colitis. Ann Pharmacother.

[7] Inoue T, Murano M, Narabayashi K, Okada T, Nouda S, Ishida K (2013). The efficacy of oral tacrolimus in patients with moderate/severe ulcerative colitis not receiving concomitant corticosteroid therapy. Intern Med.

[8] Zhang Y, Benet LZ (2001). The gut as a barrier to drug absorption: combined role of cytochrome P450 3A and P-glycoprotein. Clin Pharmacokinet.

[9] Saeki T, Ueda K, Tanigawara Y, Hori R, Komano T (1993). Human P-glycoprotein transports cyclosporin A and FK506. J Biol Chem.

[10] Hesselink DA, Van Schaik RH, Van der Heiden IP, Van der Werf M, Gregoor PJ, Lindemans J (2003). Genetic polymorphisms of the CYP3A4, CYP3A5, and MDR-1 genes and pharmacokinetics of the calcineurin inhibitors cyclosporine and tacrolimus. Clin Pharmacol Ther.

[11] Kuehl P, Zhang J, Lin Y, Lamba J, Assem M, Schuetz J (2001). Sequence diversity in CYP3A promoters and characterization of the genetic basis of polymorphic CYP3A5 expression. Nat Genet.

[12] Hirai F, Takatsu N, Yano Y, Satou Y, Takahashi H, Ishikawa S (2014). Impact of CYP3A5 genetic polymorphisms on the pharmacokinetics and short-term remission in patients with ulcerative colitis treated with tacrolimus. J Gastroenterol Hepatol.

[13] Truelove SC, Witts LJ (1955). Cortisone in ulcerative colitis. Br Med J.

[14] Ogata H, Matsui T, Nakamura M, Iida M, Takazoe M, Suzuki Y (2006). A randomised dose finding study of oral tacrolimus (FK506) therapy in refractory ulcerative colitis. Gut.

[15] Ogata H, Kato J, Hirai F, Iida M, Takazoe M, Suzuki Y (2012). Double-blind, placebo-controlled trial of oral tacrolimus (FK506) in the management of hospitalized patients with steroid-refractory ulcerative colitis. Inflamm Bowel Dis.

[16] Elens L, Bouamar R, Hesselink DA, Haufroid V, Van der Heiden IP, Van Gelder T (2011). A new functional CYP3A4 intron 6 polymorphism significantly affects tacrolimus pharmacokinetics in kidney transplant recipients. Clin Chem.

[17] Rutgeerts P, Sandborn WJ, Feagan BG, Reinisch W, Olson A, Johanns J (2005). Infliximab for induction and maintenance therapy for ulcerative colitis. N Engl J Med.

[18] Sandborn WJ, Colombel JF, D’Haens G, Van Assche G, Wolf D, Kron M (2013). One-year maintenance outcomes among patients with moderately-to-severely active ulcerative colitis who responded to induction therapy with adalimumab: subgroup analyses from ULTRA 2. Aliment Pharmacol Ther.

